# The Determinants of the Low COVID-19 Transmission and Mortality Rates in Africa: A Cross-Country Analysis

**DOI:** 10.3389/fpubh.2021.751197

**Published:** 2021-10-21

**Authors:** Yagai Bouba, Emmanuel Kagning Tsinda, Maxime Descartes Mbogning Fonkou, Gideon Sadikiel Mmbando, Nicola Luigi Bragazzi, Jude Dzevela Kong

**Affiliations:** ^1^Chantal BIYA International Reference Center for Research on HIV/AIDS Prevention and Management (CIRCB), Yaoundé, Cameroon; ^2^Department of Experimental Medicine, University of Rome “Tor Vergata”, Rome, Italy; ^3^Graduate School of Medicine, University of Tohoku, Sendai, Japan; ^4^UFR IM2AG, Université Grenoble Alpes, Grenoble, France; ^5^Graduate School of Life Sciences, University of Tohoku, Sendai, Japan; ^6^Laboratory for Industrial and Applied Mathematics (LIAM), Department of Mathematics and Statistics, York University, Toronto, ON, Canada; ^7^Africa-Canada Artificial Intelligence and Data Innovation Consortium (ACADIC), Department of Mathematics and Statistics, York University, Toronto, ON, Canada

**Keywords:** COVID-19, transmission, mortality, Africa, cross-country analysis

## Abstract

**Background:** More than 1 year after the beginning of the international spread of coronavirus 2019 (COVID-19), the reasons explaining its apparently lower reported burden in Africa are still to be fully elucidated. Few studies previously investigated the potential reasons explaining this epidemiological observation using data at the level of a few African countries. However, an updated analysis considering the various epidemiological waves and variables across an array of categories, with a focus on African countries might help to better understand the COVID-19 pandemic on the continent. Thus, we investigated the potential reasons for the persistently lower transmission and mortality rates of COVID-19 in Africa.

**Methods:** Data were collected from publicly available and well-known online sources. The cumulative numbers of COVID-19 cases and deaths per 1 million population reported by the African countries up to February 2021 were used to estimate the transmission and mortality rates of COVID-19, respectively. The covariates were collected across several data sources: clinical/diseases data, health system performance, demographic parameters, economic indicators, climatic, pollution, and radiation variables, and use of social media. The collinearities were corrected using variance inflation factor (VIF) and selected variables were fitted to a multiple regression model using the R statistical package.

**Results:** Our model (adjusted R-squared: 0.7) found that the number of COVID-19 tests per 1 million population, GINI index, global health security (GHS) index, and mean body mass index (BMI) were significantly associated (*P* < 0.05) with COVID-19 cases per 1 million population. No association was found between the median life expectancy, the proportion of the rural population, and Bacillus Calmette–Guérin (BCG) coverage rate. On the other hand, diabetes prevalence, number of nurses, and GHS index were found to be significantly associated with COVID-19 deaths per 1 million population (adjusted R-squared of 0.5). Moreover, the median life expectancy and lower respiratory infections rate showed a trend towards significance. No association was found with the BCG coverage or communicable disease burden.

**Conclusions:** Low health system capacity, together with some clinical and socio-economic factors were the predictors of the reported burden of COVID-19 in Africa. Our results emphasize the need for Africa to strengthen its overall health system capacity to efficiently detect and respond to public health crises.

## Introduction

The global pandemic of “coronavirus disease 2019” (COVID-19), caused by the “Severe acute respiratory syndrome coronavirus-2” (SARS- CoV-2) first emerged in Wuhan City, Hubei Province of China (1–3). The SARS-CoV-2 infection rapidly spread in China and thereafter, was reported in the United States, Europe, and all the other continents (4–7). Following this international spread and the threat it represents for the world, the Director-General of the WHO declared COVID-19 a “global pandemic” on 11 March 2020.

Regarding the African continent, the first case, a contact of a person with a history of travel to China was notified by Egypt, an African country predicted to be among those with a high importation risk, on 14 February 2020 (7, 8). The epidemic evolved rapidly and as of 15 March 2020, a total of 25 countries reported COVID-19 cases in the WHO AFRO region (https://apps.who.int/iris/bitstream/handle/10665/331451/OEW11-0915032020.pdf). In this regard, the WHO has called Africa to “wake up” to the coronavirus threat and prepare for the worst scenario. There was an agreement among nearly all the predictive models that the new pandemic will be disastrous on the African countries, which have a weaker health system and are already facing huge public health challenges; this would be in terms of transmission, severity, and impact of COVID-19 (9–14).

Though the COVID-19 pandemic hit, directly and indirectly, the vital health services on the continent (15), unexpectedly, Africa has not yet experienced the predicted large burden of COVID-19 (16–18). As of 4 September 2021, the entire continent has reported a toll of approximately 8 million cases and 200,000 deaths, 3.6% and 4.4% of the global figures, respectively, despite the African population is about 16% of the worldwide population.

A number of hypotheses have been proposed to explain this unexpected observation. The early response and continent wide-response, under-reporting, younger age of the population, low international air traffic flows, the climate, cross-immunity, and the use of anti-parasitic drugs are among the commonest listed factors (7, 19–23). Additional factors, such as the effect of Bacillus Calmette–Guérin (BCG) vaccination, genetic background, population demography, climatic factors, and economic factors have been previously investigated to explore their association with COVID-19 burden (24–29).

So far, most of these studies analyzed only a single or few groups of possible covariates, but the factors determining the patterns of COVID-19 transmission, as it is the case of many other emerging/re-emerging infectious diseases, require a multi-factorial analysis. To the best of our knowledge, no study investigated the association of biological, clinical, organizational (health system capacity), economical-financial, climatic, environmental (including pollution, radiation, and air traffic) and behavioral (use of social media) parameters focusing only on the African countries, which, on the one hand, share among themselves similarities, but, on the other hand, are uniquely different from the Western countries, with potential country-specific confounders that might not be adjusted for in most models.

Moreover, an updated analysis taking into consideration the various epidemiological waves that occurred on the continent might provide public health decisions- and policymakers with useful information to properly deal with the still ongoing pandemic. To overcome some of the methodological gaps outlined above, in this analysis specifically focused on Africa, we investigated all the factors potentially related to COVID-19 burden, to obtain a comprehensive picture of the plausible reasons for the observed persistently lower transmission and mortality rates of COVID-19 on the African continent, more than a year after the first cases were reported on the continent.

## Materials and Methods

### Aim and Design of the Study

This study aimed to investigate the factors associated with the low burden of COVID-19 in Africa. More specifically, we investigated the factors potentially connected with the risk of COVID-19 transmission (number of COVID-19 cases per 1 million population) and the risk of COVID-19 fatality (number of COVID-19 deaths per 1 million population). To achieve this objective, data concerning different categories of covariates (epidemiological, socio-demographic, climatic, environmental, and economical-financial) from 54 African countries were collected and the multiple linear regression model was fitted to the data.

## Data Collection

### SARS-CoV-2 Transmission and Mortality

The data for SARS-CoV-2 transmission and mortality came from the coronavirus page of “Our World in Data” database (github.com/owid/covid-19-data/tree/master/public/data). The cumulative number of COVID-19 reported cases in the African countries from the beginning of the pandemic on February 14, 2020, to February 4, 2021, were extracted. Given that we aimed to investigate both COVID-19 transmission risk and fatality, we used two outcome variables separately to construct the models. To estimate COVID-19 transmission, we chose the total confirmed cases of COVID-19 per 1 million populations, over the total absolute number of cases because it is a better proxy to estimate the risk of being infected. Similarly, the total deaths attributed to COVID-19 per 1 million populations were used to estimate the risk of dying, which is a measure of disease severity.

### Independent Variables

The covariates used in the analysis were collected across several fields of science, including clinical/diseases data, health system performance, demographic parameters, economic indicators, climatic variables, the levels of pollution and radiation, and the use of social media. The data were collected from publicly available and well-known, reliable sources, such as the World Bank Open Data, the Health Nutrition and Population Statistics, the WHO Global Health Observatory (GHO), the Institute of Health Metrics and Evaluation (IHME), the Global Health Security Index (GHS), and the Our World in Data databases among the others. The description of each covariate by category is reported in Table 1. These variables were included based on the list of variables previously hypothesized to be among the possible reasons for the low burden of COVID-19 in Africa. These variables have been reviewed in two scientific literature produced by our research group (one of them is currently under peer-review) (30).

**Table 1:** List of independent variables collected from various online sources.

**Factors**	**Description, measure/unit**	**Source**
**Demographic**		
*Total population*	Total number of the population, 2020 data	population.un.org/wpp/Download/Standard/Population/
*Life expectancy*	Median year of life expectancy at birth, in years	github.com/owid/covid-19-data/tree/master/public/data
*Population density*	Number of people by land area, measured in square kilometers, most recent year available	github.com/owid/covid-19-data/tree/master/public/data
*Median age*	Median age of the population, in years	github.com/owid/covid-19-data/tree/master/public/data
*Rural population*	Proportion of the population living in rural area, % of total population	databank.worldbank.org/source/health-nutrition-and-population-statistics
*Urbanization*	Population in urban agglomeration of more than 1 million, % of total population	data.worldbank.org/indicator/EN.URB.MCTY.TL.ZS
**Clinical or diseases**		
*Cardiovascular diseases*	Death rate due to cardiovascular diseases, per 100,000 population	github.com/owid/covid-19-data/tree/master/public/data
*Diabetes*	Diabetes prevalence (% of population ages 20 to 79)	data.worldbank.org/indicator/SH.STA.DIAB.ZS
*TB*	Incidence rate of TB, per 100,000 people	data.worldbank.org/indicator/SH.TBS.INCD
*BCG*	BCG vaccination coverage, in %	databank.worldbank.org/source/health-nutrition-and-population-statistics
*LRI*	Lower respiratory infections rate, per 100 000 population	ghdx.healthdata.org/gbd-results-tool
*HIV*	Prevalence of HIV, total (% of population ages 15-49)	databank.worldbank.org/source/health-nutrition-and-population-statistics
*Malaria*	Reported cases of malaria, absolute number	databank.worldbank.org/source/health-nutrition-and-population-statistics
*BMI*	Mean body mass index of 18+ years, in Kg/m^2^	https://www.who.int/data/gho/data/indicators/indicator-details/GHO/mean-bmi-(kg-m-)-(crude-estimate)
*Raised BP*	Raised blood pressure (SBP≥140 OR DBP≥90), age-standardized estimate	https://www.who.int/data/gho/data/indicators/indicator-details/GHO/raised-blood-pressure-(sbp-=140-or-dbp-=90)-(age-standardized-estimate)
*Raised cholesterol*	Raised total cholesterol (≥ 5.0 mmol/L), age-standardized estimate	https://www.who.int/data/gho/data/indicators/indicator-details/GHO/raised-total-cholesterol-(-=-5-0-mmol-l)-(age-standardized-estimate)
*Communicable diseases*	*Burden of* communicable diseases and maternal, prenatal and nutrition conditions (include infectious and parasitic diseases, respiratory infections), per 100 000 populations	ghdx.healthdata.org/gbd-results-tool
*Cancer*	Cancer prevalence, in %	ourworldindata.org/grapher/share-of-population-with-cancer?tab=table
**Epidemiological**		
Transmission level	WHO transmission classification (community transmission versus no community transmission	covid19.who.int/table
**Health system related**		
Testing capacity	Total tests for COVID-19 per one million population	population.un.org/wpp/Download/Standard/Population/
*Nurses*	Number of nurses and midwives per 1000 population	data.worldbank.org/indicator/SH.MED.NUMW.P3
*Physician*	Number of physicians per 1000 population	data.worldbank.org/indicator/SH.MED.PHYS.ZS
*GHS index*	Global health security detection index (Weighted sum of all the GHS data normalized to a scale of 0 to 100, where 100 = best health security condition)	https://www.ghsindex.org/
**Socio-economic**		
GDP	Gross domestic product per capita	github.com/owid/covid-19-data/tree/master/public/data
*GINI Index*	GINI index (income inequality, 100 = high	github.com/owid/covid-19-data/tree/master/public/data
*Doing business*	Ease of doing business rank 2019 (1=most business-friendly regulations)	databank.worldbank.org/source/doing-business#selectedDimension_Country
*Human development index*	Human development index	ourworldindata.org/human-development-index
**Climatic**		
Temperature	Temperature in degree Celsius, annual mean	https://tinyurl.com/d3pnyu9m
*Rainfall*	Rainfall in mm, annual mean	tinyurl.com/d3pnyu9m
**Pollution**		
Pollution	PM2.5 air pollution, mean annual exposure (micrograms per mm^3^)	data.worldbank.org/indicator/EN.ATM.PM25.MC.M3
*UV radiation*	Ultraviolet radiation exposure	
**Traffic**		
Air traffic	Air transport, passengers carried per capita	https://data.worldbank.org/indicator/IS.AIR.PSGR
**Social media use**		
Internet filtering	Government internet filtering in practice (4 = low)	digitalsocietyproject.org/data/

*BCG, Bacille Calmette et Guérin; BMI, body mass index; BP, blood pressure; GDP, gross domestic product; GHS, global health security; LRI, lower respiratory tract infections; TB, tuberculosis*.

### Statistical Analysis

We summarized continuous variables as means and SDs, while the qualitative/categorical variables were expressed as proportions. Few missing values were found for 15 variables and imputed with median value since the median is robust to outliers. The “variance inflation factor” (VIF) was used to measure how much the variance of the estimated regression coefficient was increased due to collinearity and variables with a VIF greater than 10 were removed from the analysis. The numerical variables were scaled to ease downstream comparison of regression coefficients.

The selected covariates were used to analyze the relationship between the dependent and independent variables while controlling for other epidemiological, economical/financial, and organizational (health system) related indicators. To determine which covariates were significantly associated with COVID-19 transmission and death risk in Africa, the number of COVID-19 cases per 1 million population and the number of COVID-19 deaths per 1 million population were considered as our response variables, respectively. The sign and significance of the regression model coefficients were used to assess the effects of the independent variables on the outcome variable. After compiling the variables, they were then fitted to a multiple regression model using the “lm” function of the “stats” package of the R statistical environment, version 3.5 (R Core Team 2020, Austria).

The general formula of the multiple linear regression model is given as follows:


$$\begin{array}{l} y\;=\;v_{0}+v_{1}x_{1}+v_{2}x_{2}+\cdots +v_{n}x_{n} \end{array}$$

Where “*y*” is the dependent or outcome variable; *v*_0_, *v*_1_, *v*_2_, …, *v*_*n*_ are the unknown parameters estimated by the regression model; and *x*_1_, *x*_2_, *x*_3_, …, *x*_*n*_ are independent covariates variables. The *P*-values less than 0.05 were considered statistically significant.

### Radar Plot

To visualize the scaled values of statistically significant covariates from the regression model, the African countries having a wide range of COVID-19 cases per 1 million populations and COVID-19 deaths per one million populations were represented on a radar chart, constructed using Microsoft Office Excel 2016 (Microsoft Corporation, WA, USA).

## Results

### Regression Analysis Results

The results obtained from fitting the variables in Table 1 to a multiple regression model are shown in Tables 2, 3, for the cases per 1 million populations and deaths per 1 million populations, respectively.

**Table 2:** Multiple regression analysis using COVID-19 cases per one million populations as the dependent variable.

**Covariates**	**Estimate**	**Standard error**	***t*-value**	***p*-value**
COVID-19 Community transmission	3839.7	2396.1	1.603	0.1216
Number of COVID-19 tests per one million population	**3300.5**	**1260.9**	**2.618**	**0.0148**
Number of populations	−806.1	991.2	−0.813	0.4237
Median life expectancy	1674.7	1155.3	1.45	0.1596
Proportion of rural population	168.5	1406.5	0.12	0.9056
Proportion living in urban agglomeration	394.2	796.3	0.495	0.6249
Gross domestic product per capita	−1030.7	1553	−0.664	0.5129
GINI index	**1959.7**	**834.2**	**2.349**	**0.0270**
Doing business rank	1814.8	1110	1.635	0.1146
Cardiovascular diseases rate	−1139.3	794.1	−1.435	0.1638
Diabetes prevalence	−1398.3	1147.4	−1.219	0.2343
Tuberculosis incidence	157	960.7	0.163	0.8715
BCG coverage	−350	797.2	−0.439	0.6644
Lower respiratory infection rate	529.7	767.5	0.69	0.4964
Number of nurses	−2136.6	1242.4	−1.72	0.0978^*^
Number of physicians	−682.9	1235.9	−0.553	0.5855
GHS index	**1821.1**	**854.1**	**2.132**	**0.0430**
Mean BMI	**2794**	**1235.4**	**2.262**	**0.0327**
Number of malaria cases	813.2	945.2	0.86	0.3978
Communicable diseases burden^†^	−1306.6	1612	−0.811	0.4253
Cancer prevalence	2824.4	1506	1.875	0.0725^*^
Raised BP	244.5	1386.3	0.176	0.8614
Air traffic	1207.7	1335.5	0.904	0.3745
Ultraviolet radiation	−291.5	1221.6	−0.239	0.8133
PM2.5 air pollution exposure	128.9	732.7	0.176	0.8617
Annual mean temperature	−253.3	823	−0.308	0.7608
Annual mean rainfall	−913.6	1069.5	−0.854	0.4011
Internet filtering	−424.1	887.3	−0.478	0.6368

*Bold character indicates statistical significance (P < 0.05)*.

* *Indicates variables with a trend towards statistical significance (0.05 < p < 0.10)*.

† *Communicable diseases and maternal, prenatal, and nutrition conditions. BCG, Bacille Calmette et Guérin; BMI, body mass index; BP, blood pressure, defined as SBP ≥ 140 OR DBP ≥ 90; GHS, global health security*.

**Table 3:** The multiple regression analysis outputs using COVID-19 deaths per one million populations as a dependent variable.

**Covariates**	**Estimate**	**Standard Error**	***t*-value**	***p*-value**
(Intercept)	−1254.98	2591.06	−0.484	0.6324
COVID-19 community transmission	3436.51	2891.65	1.188	0.2458
Number of tests per one million population	−109.08	1531.39	−0.071	0.9438
Number of populations	561.4	1319.94	0.425	0.6742
Life expectancy	2961.46	1563.74	1.894	0.0699^*^
Proportion of rural population	−3309.14	2006.25	−1.649	0.1116
Proportion living in urban agglomeration	−673.02	1057.79	−0.636	0.5304
Gross domestic product per capita	−1718.18	2147.68	−0.8	0.4312
GINI index	1433.11	1239.62	1.156	0.2586
Doing business rank	677.27	1482.61	0.457	0.6518
Diabetes prevalence	**2923.41**	**1407.29**	**2.077**	**0.0482**
Tuberculosis incidence rate	−1044.7	1488.99	−0.702	0.4894
BCG coverage	−333.45	1073.33	−0.311	0.7586
Lower respiratory infection rate	1873.49	1034.67	1.811	0.0822^*^
HIV prevalence	2782.98	1911.04	1.456	0.1578
Number of nurses	**−3821.9**	**1653.49**	**−2.311**	**0.0293**
Number of physicians	−197.2	1763.89	−0.112	0.9119
GHS index	**2871.86**	**1127.17**	**2.548**	**0.0174**
Mean BMI	1050.76	1636.1	0.642	0.5266
Number of malaria cases	−12.97	1248.88	−0.01	0.9918
Communicable diseases burden^†^	3543.01	2161.26	1.639	0.1137
Cancer prevalence	1540.17	1978.97	0.778	0.4437
raised BP	−272.75	1808.56	−0.151	0.8813
Air traffic	1193.02	1770.7	0.674	0.5067
Ultraviolet radiation	−2083.54	1535.16	−1.357	0.1868
PM2.5 air pollution exposure	33.07	1058.29	0.031	0.9753
Annual mean temperature	524.94	1103.41	0.476	0.6384
Annual mean rainfall	−1251.68	1398.24	−0.895	0.3792
Internet filtering	−1083.98	1196.02	−0.906	0.3734

*Bold character indicates statistical significance (P < 0.05)*.

* *Indicates variables with a trend towards statistical significance (0.05 < p <0.10)*.

† *Communicable diseases and maternal, prenatal, and nutrition conditions. BCG, Bacille Calmette et Guérin; BMI, body mass index; BP, blood pressure, defined as SBP ≥ 140 OR DBP ≥ 90; GHS, global health security*.

### Factors Associated With COVID-19 Cases per 1 Million Populations in Africa

The regression model constructed to determine the factors associated with COVID-19 cases per 1 million populations in Africa had an adjusted R-squared of 0.7 and the variables significantly associated (*P* < 0.05) with COVID-19 cases per 1 million populations on the African continent were the number of COVID-19 tests per 1 million populations, the GINI inequality index, the GHS detection index, and the mean body mass index (BMI). Table 2 shows, for example, that a unit increase in COVID-19 tests per 1 million populations, GINI index, the GHS, and BMI were significantly associated with nearly 3,300, 1,960, 1,821, and 2,794 units increase of the COVID-19 cases per 1 million population in Africa, respectively. Also, the number of nurses per 1,000 populations and cancer prevalence showed a negative and positive trend towards significance, respectively. Of note, no association was found with the median life expectancy, the proportion of the rural population, prevalence of diabetes, rate of cardiovascular diseases, BCG coverage, number of reported malaria cases, and climate variables.

### Factors Associated With COVID-19 Deaths per 1 Million Population in Africa

The second regression model shown in Table 3, constructed to determine factors associated with COVID-19 deaths per 1 million populations in Africa had an adjusted R-squared of 0.5 and the variables significantly associated (*P* < 0.05) with COVID-19 deaths per 1 million populations on the African continent are the prevalence of diabetic patients, number of nurses per 1,000 populations, and the GHS detection index. This table shows that a unit increase in the prevalence of diabetic patients, number of nurses per 1,000 populations, and the GHS index was significantly associated, respectively with 2,923, 3,821, and 2,871 units increase in the COVID-19 deaths per 1 million populations in Africa. Therefore, the rate of diabetics and health system related indices (nurses, GHS) are important predictors of COVID-19 fatality in the people of Africa. Moreover, the median life expectancy and lower respiratory infections rate showed a trend towards significance. Of note, no associations between COVID-19 deaths per 1 million populations and BCG coverage, number of reported malaria cases, or communicable disease burden were found.

### Graphical Representation of Significantly Associated Variables

**Figure 1:**
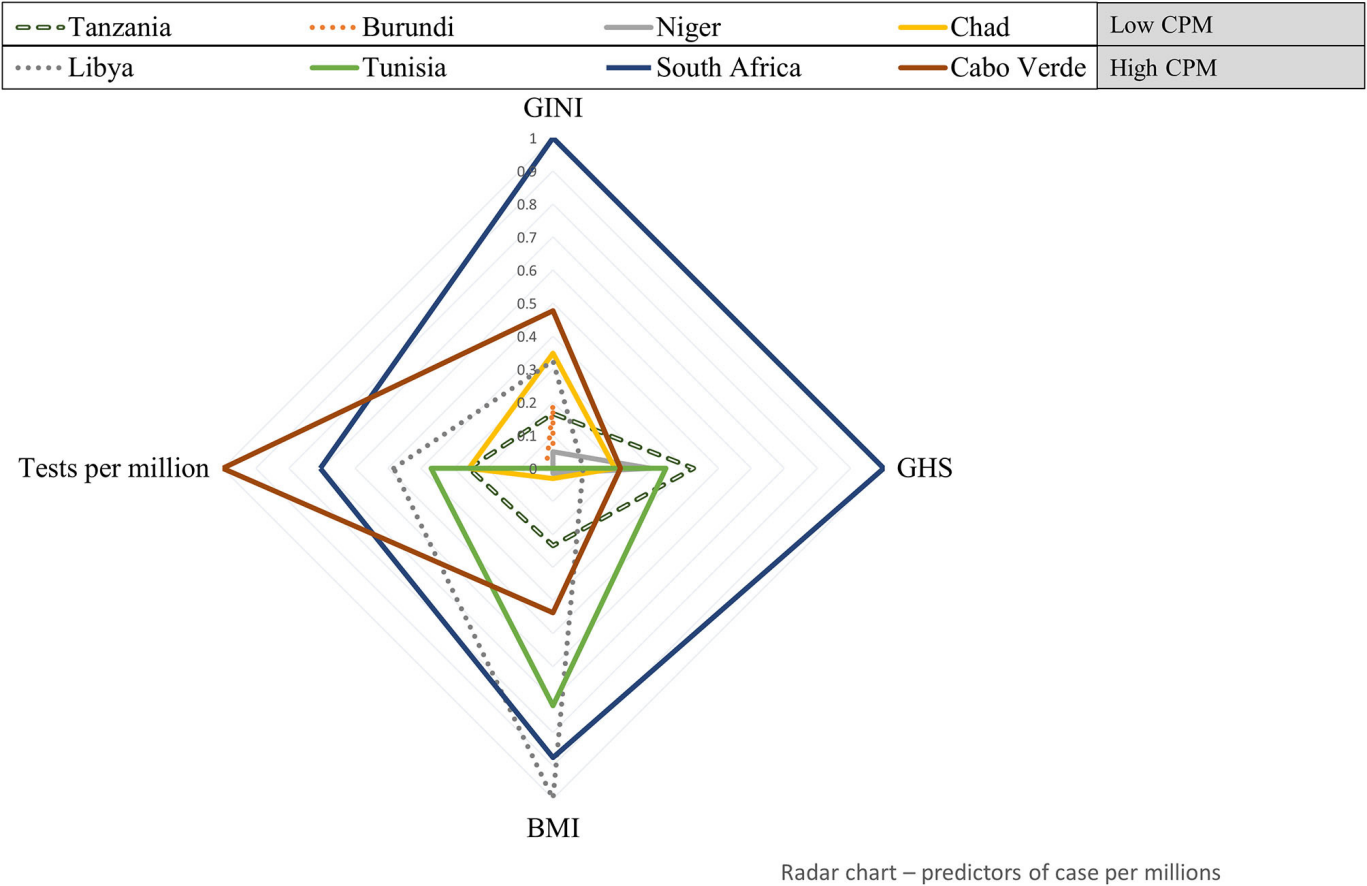
Health system, socio-economic, and clinical factors' profile of eight African countries with a wide range of COVID-19 cases per 1 million populations. Standardized values of variables significantly associated with COVID-19 cases per 1 million populations (test per millions, GINI, global health security [GHS], and body mass index [BMI]) are plotted for eight countries having the lowest **(upper-left)** to highest **(lower-right)** COVID-19 cases per million. CPM, Cases per 1 million population.

Figure 1 shows how eight countries having a wide range of COVID-19 cases per 1 million populations, with scaled values representing the number of tests per 1 million populations are characterized by the GINI inequality index, GHS index, and BMI. This radar plot shows that the country ranking in covariate values mostly conforms to the statistical trends suggested by the regression model. For example, when compared with the countries having high COVID-19 cases per 1 million populations, e.g., South Africa, Tanzania, Burundi, Nigeria, and Chad, which have the lowest COVID-19 cases per 1 million populations display a lower number of tests per 1 million populations, a lesser BMI, lower GINI inequality index, and lesser GHS index. This indicates that most countries with a high number of COVID-19 cases per million populations tend to fit the statistically high COVID-19 cases profile suggested by the regression model. However, some exceptions are observed. This is the case of Tunisia which shows a relatively low number of tests per 1 million populations and a very low GINI index but is among the countries with the highest transmission rate.

**Figure 2:**
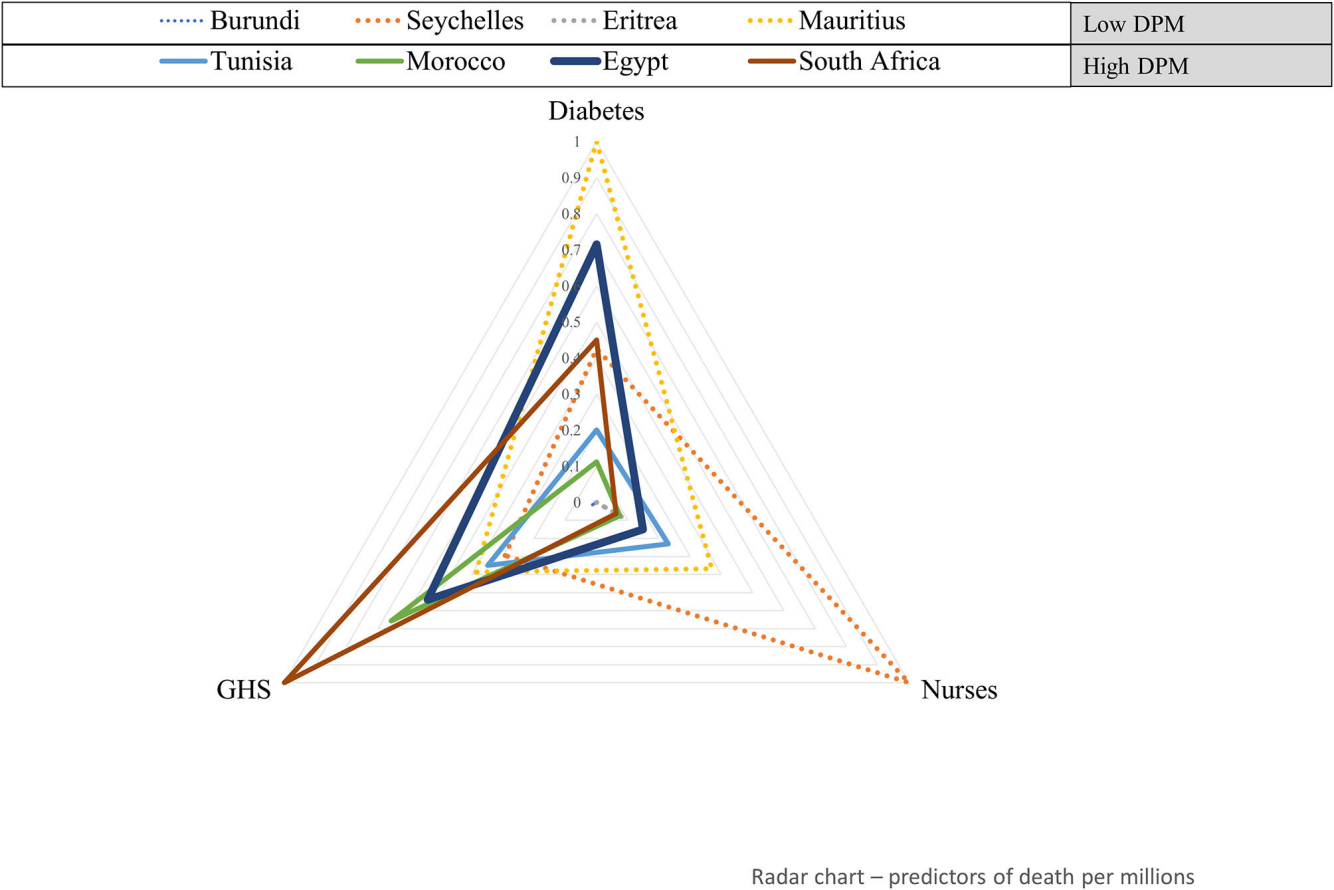
Health system and clinical factors' profile of eight African countries with a wide range of COVID-19 deaths per 1 million populations. Standardized values of variables significantly associated with COVID-19 deaths per 1 million populations (GHS index and diabetes) are plotted for eight countries having the lowest **(upper-left)** to highest **(lower-right)** COVID-19 DPM. DPM, Deaths per one million population.

Figure 2 shows how eight countries covering a wide range of COVID-19 deaths per 1 million populations are characterized by the prevalence of diabetes, number of nurses, and the GHS index. This radar plot shows that the country ranking in the covariate values conforms in part with the findings from the regression model. In general, when compared with countries with the lowest number of COVID-19 deaths per 1 million populations, South Africa, Egypt, and Tunisia have a higher prevalence of diabetes and GHS index. An opposite trend was observed for the number of nurses per 1000 populations which had a negative association in the model. As was the case with transmission rate, exceptions exist; Mauritius, for example, has a low rate of COVID-19 deaths but recorded a high prevalence of diabetes.

## Discussion

The COVID-19 pandemic has been a devastating crisis worldwide and more especially in Western countries. Despite the availability of several preventive measures, including the vaccine roll-out and the huge body of scientific knowledge generated to understand its pathophysiology and its transmission patterns, it still remains one of the major global health concerns. In this analysis with a focus on Africa, we investigated the factors with potential effects on the risk of COVID-19 infection and severity (mortality rate).

The variables that independently predicted the risk of acquiring COVID-19 infection were socio-economic factors, clinical factors, and health system capacity to detect and respond to epidemics. Some authors argue that the low burden of COVID-19 might be explained by the flaws in the detection and reporting system of most African countries (31). Our model constructed on data collected on African countries confirmed that the number of tests performed per million population was positively and significantly associated with the number of cases per million population (*P* = 0.0148). It is not excluded that this association can also be explained by the fact that the countries with high testing (likely due to high demand in testing) would be likely those with a high risk of infection transmission. However, this finding was further supported by the fact that the number of cases per million population was predicted by the GHS detection index (*P* = 0.0430), a measure of the overall health security and related capabilities (24). Though the COVID-19 pandemic has revealed the underestimation of the preparedness of some countries with lower scores on the GHS index (32), efforts are needed by African countries to improve their overall health system capacity, including GHS (33), which ranges from 55% (South Africa) to 16% (Equatorial Guinea) (Supplementary Table 1).

In the same line, we found that the higher the number of nurses and midwives per 1,000 population, the lower the risk of COVID-19 infection (even though only borderline statistically significant, *P* = 0.0978). On the other hand, the lower median age of the population in Africa (<20 years) has been most often found to be associated with the decreased severity of symptoms of COVID-19 infection on the continent. Several reports indicate that, in general, the adult population (>20 years) contributes disproportionately to the worldwide number of COVID-19 cases (34–36). However, our results suggest that life expectancy was not an independent predictor of the risk of COVID-19. This might imply that the detection of COVID-19 infection should be extended across all age groups for better control of the pandemics.

Another important finding was that the risk of COVID-19 infection increased with the inequalities index (GINI) within a country (*P* = 0.0270). This could be explained by the fact that high GINI values lead to the presence of more vulnerable people, generally working in the informal sector; and thus, to higher exposure to COVID-19. This is especially true for Africa where inequalities are fueled by conflicts and instabilities. Such situations might lead to limited access to healthcare facilities and to a higher number of infectious diseases, including the COVID-19 infection (24).

Interestingly, we found that the mean BMI in the adult population was positively associated with the risk of acquiring COVID-19 infection (*P* = 0.0327). The association of BMI with an increased risk of COVID-19 infection was previously documented in the literature (37, 38). Obesity and unhealthy lifestyle in general increase the risk of diseases, achieved through the impairment of immunity (39). Given that a study showed that an increased BMI was especially observed in people younger than 40 yr and of Black ethnicity (40), an appropriate policy to control the risk of infection in this group is needed. Our analysis did not confirm the increased risk with other underlying comorbidities, such as diabetes, cardiovascular diseases, and hypertension (41, 42). This analysis equally confirms that particular attention should be given to the patients with cancer, which, even though only with a trend, was associated with increased COVID-19 infection (*P* = 0.0725). Such association was previously documented and was especially attributed to lung cancer (43).

Of note, some clinical factors previously documented to be associated with the increased risk of COVID-19 infection, which was not confirmed in our analysis are as follows BCG vaccination coverage (44–47); the number of reported malaria cases (48–55), and deaths due to communicable diseases (56, 57). Lastly, regarding the environmental factors, such as rainfall, temperature, and exposure to UV radiation, contrary to some reports, we did not observe any association (23, 58).

Regarding the fatality of COVID-19 (deaths per 1 million population), our model indicates that about 50% of the risk of dying was explained by the variables we considered (Adjusted R-squared: 0.4976, 0.00468). Among the variables significantly associated are the clinical, health system related, and demographic factors.

Regarding the clinical factors, our data confirmed that the prevalence of diabetes independently predicted the risk of death. This observation has been widely documented also at the individual level (59, 60). This has been attributed to the fact that the patients with diabetes have delayed viral clearance, decreased immunity, and increased angiotensin-converting enzyme 2 (ACE2) expression, leading to an inappropriate response to COVID-19 infection and other secondary infections (41, 61, 62). Another consideration is that older age, which is a risk of COVID-19 severity is also associated with diabetes, with its prevalence and underlying complications increasing with older age (63, 64). Indeed, we found that life expectancy, which is a proxy of the proportion of the older age, slightly predicted the number of deaths per million. Another clinical factor found to be slightly associated with the COVID-19 deaths was the mortality rate due to the lower respiratory infections (*P* = 0.0822). This finding was also expected because a systematic review and meta-analysis reported that COVID-19 co-infections or superinfections with respiratory pathogens were associated with an increased odds of death (65). Of note, the BCG coverage, the number of malaria cases, and the burden of communicable diseases did not seem to have any protective effects on the number of deaths per million (*P* < 0.05 for all). Contrary to the risk of being infected, the GINI index was not associated with COVID-19 severity. This is probably because the population in Africa is generally very young (median age: 20 years), thus the severity will be less, likely even in the situations of promiscuity and precarity. This homogeneous population is also demonstrated by the fact that the rural population and those living in highly populated cities were not associated with COVID-19 death.

Concerning the health system related factors, the number of nurses (*P* = 0.0293) and the GHS index (*P* = 0.0174) positively predicted the COVID-19 fatality, as it was the case of the risk for acquiring the infection. The response capacity of a health system is indeed important to respond to COVID-19 infection, especially in elderly people and those with comorbidities, since their clinical management requires hospitalization. The insufficient number of nurses, in particular, would affect the access to healthcare facilities and the quality of healthcare provided.

Our findings have practical implications in terms of the achievement of herd immunity. Intriguingly, median life expectancy showed a trend towards statistical significance. COVID-19 spread among the youth may result in a higher number of asymptomatic/mild cases, with fewer cases, making populations not immunologically naïve to the virus anymore. This should be verified by seroepidemiologic surveys (66).

In conclusion, our model suggests that there are many explanatory variables that account for the risk of COVID-19 infection and death in Africa. These may span across the biological and epidemiological variables, including the growth rate and the basic reproduction number of the virus (67, 68), logistic-organizational (69, 70), and socio-cultural factors. The COVID-19 pandemic represents, both directly and indirectly, a threat for the continent, thus Africa should continue to strengthen its health system, also by building on its previous experience with infectious outbreaks (such as Ebola or Zika) to mitigate against the effects of the still ongoing pandemic.

However, our study has some limitations. First, as for all the ecological investigations, our study is subject to “ecological fallacy” and biases. For example, some of the data analyzed are not up-to-date, and therefore might not reflect the current situation. This is especially true for non-communicable diseases that rapidly rise in Africa. Second, there might also be some biases in the reporting of the number of cases and deaths, due to the different testing and reporting capacity across the African countries, and biases in the measurement and reporting of some covariates, such as the GINI index, which was expected to be high in some countries with instability and security issues, such as Chad. Third, the variables associated with COVID-19 cases and deaths reported in this study do not necessarily imply causality as this study design is not suitable for drawing any causal relationship. Another study limitation is the relatively moderate R-squared value (especially of the second model), which suggests that other variables not included in our regression model may explain from 35 to 40% of the variance in the number of COVID-19 cases and deaths per million population.

## Conclusions

In the present study, we found that in Africa, the clinical conditions and health system capacity were predictors of both COVID-19 transmission and fatality, while socio-economic indices were associated only with the risk of COVID-19 transmission. This study complements and adds to the existing studies investigating the possible reasons for the low rates of COVID-19 cases and deaths in Africa and offering a broader picture at the continent level.

Our results emphasize the need for Africa to strengthen its overall health system capacity to efficiently detect and respond to COVID-19, as well as to potential future pandemics and outbreaks. Further studies based on the data collected at an individual level are warranted to confirm these observations. Moreover, for a better understanding of COVID-19 epidemiology across the continent, such studies should also consider the SARS-CoV-2 viral diversity.

## Data Availability Statement

Publicly available datasets were analyzed in this study. This data can be found here: github.com/Jdkong/socioeconomicafrica.

## Author Contributions

All the authors contributed to the conception and development of the study. YB, ET, MF, and GM collected and prepared the data. YB, NB, JK, ET, and MF analyzed the data. All the authors contributed significantly to the drafting of the manuscript and approved its final version. JK and NB revised the manuscript.

## Funding

This research is funded by the International Development Research Center (IDRC), Canada (Grant No. 109559-001).

## Conflict of Interest

The authors declare that the research was conducted in the absence of any commercial or financial relationships that could be construed as a potential conflict of interest.

## Publisher's Note

All claims expressed in this article are solely those of the authors and do not necessarily represent those of their affiliated organizations, or those of the publisher, the editors and the reviewers. Any product that may be evaluated in this article, or claim that may be made by its manufacturer, is not guaranteed or endorsed by the publisher.

## Supplementary Material

Supplementary Table 1 Descriptive statistics of the main factors of interest.
