# Supplementary material for: The Determinants of the Low COVID-19 Transmission and Mortality Rates in Africa: A Cross-Country Analysis
Source: Front Public Health. 2021 Oct 21;9:751197. doi: 10.3389/fpubh.2021.751197 (PMC8568130; doi:10.3389/fpubh.2021.751197)
Supplement: Supplementary Table 1 — Descriptive statistics of the main factors of interest. [file Table_1.docx]

**Supplementary Table 1.** Descriptive statistics of the main factors of interest.

| **Variables** | **Mean** | **SD** | **Minimum** | **Maximum** |
| --- | --- | --- | --- | --- |
| **COVID-19 cases and deaths per million** | | | | |
| *Cumulative cases per million population* | 3450.9 | 3621.0 | 8.5 | 25702.0 |
| *Cumulative deaths per million population* | 61.7 | 67.0 | 0.3 | 768.9 |
| **Socio-demographic factors** | | | | |
| *Total population* | 23833218 | 21939957 | 98340 | 206139587 |
| *Median year of life expectancy at birth (years)* | 63 | 4 | 53 | 77 |
| *Median age of the population (years)* | 20 | 3 | 15 | 37 |
| *Proportion of rural population (% of total population)* | 55.6 | 14.7 | 10.3 | 86.6 |
| *Population in urban agglomeration of more than 1 million (%)* | 16.8 | 8.4 | 4.0 | 61.3 |
| *Population density (square kilometres)* | 109.8 | 93.9 | 3.1 | 623.0 |
| **Socio-economic factors** | | | | |
| *Human development index* | 0.533 | 0.085 | 0.354 | 0.797 |
| *Gross domestic product per capita* | 4991.2 | 4367.0 | 661.2 | 26382.3 |
| *GINI index (income inequality, 100= high* | 43 | 6 | 28 | 63 |
| *Ease of DB rank 2019 (1=most business-friendly regulations)* | 138 | 32 | 13 | 189 |
| **Health system related factors** | | | | |
| *GHS detection index (scale of 0 to 100, where 100= best GHS)* | 31 | 6 | 16 | 55 |
| *Number of nurses and midwifes per 1000 population* | 1.4 | 1.0 | 0.1 | 8.1 |
| *Number of physicians per 1000 population* | 0.3 | 0.3 | 0.0 | 2.5 |
| *Number of total tests per 1 million population* | 49640 | 49107 | 3107 | 296554 |
| **Clinical or diseases factors** | | | | |
| *BCG vaccination coverage, in %* | 87.4 | 9.1 | 52.0 | 99.0 |
| *Incidence rate of TB, per 100,000 people* | 215.6 | 127.9 | 12.0 | 654.0 |
| *Diabetes prevalence (% of population ages 20 to 79)* | 5.1 | 2.4 | 1.0 | 22.0 |
| *Mean body mass index of 18+ years, in Kg/m^2^* | 24 | 1 | 21 | 27 |
| *Raised BP (SBP>=140 OR DBP>=90), % of adult population* | 28.4 | 1.9 | 23.5 | 33.4 |
| *Raised total cholesterol (>= 5.0 mmol/L), % of population* | 25.1 | 6.7 | 15.2 | 57.7 |
| *Lower respiratory infections rate, per 100 000 population* | 155.5 | 20.4 | 96.8 | 206.5 |
| *Cancer prevalence, in %* | 0.5 | 0.1 | 0.3 | 1.3 |
| *Reported cases of malaria, absolute number* | 2680134 | 2839755 | 0 | 16972207 |
| **Communicable Diseases per 100 000 populations* | 74182.7 | 5927.2 | 40417.4 | 87625.6 |
| *Death rate due to CVDs, per 100 000 population* | 273.5 | 47.5 | 181.0 | 435.7 |
| *Prevalence of HIV (% of population ages 15-49)* | 4.7 | 4.7 | 0.1 | 27.0 |
| **Environmental and internet access factors** | | | | |
| *Air transport, passengers carried per capita* | 0.3 | 0.3 | 0.0 | 4.7 |
| *Ultraviolet radiation exposure mW/cm2* | 5132 | 379 | 3253 | 5929 |
| *PM2.5 air pollution exposure, annual mean μg/mm^3^)* | 36.9 | 12.3 | 14.5 | 94.1 |
| *Temperature in degree Celsius, annual mean* | 25 | 3 | 14 | 29 |
| *Rainfall in mm, annual mean* | 85 | 40 | 5 | 224 |
| *Government internet filtering in practice (4= low)* | 3 | 1 | 1 | 4 |
| ** Communicable Diseases and maternal, prenatal and nutrition conditions. BCG: Bacille Calmette et Guérin; CVDs: cardiovascular diseases; DB: doing business; DBP: diastolic blood pressure; GHS: global health security index; SBP: systolic blood pressure; SD: standard deviation; TB: tuberculosis.* | | | | |

**Source:** author’s calculation based on the latest available data collected from publicly available sources.
